# Tasting More Than Just Food: Effect of Aesthetic Appeal of Plate Patterns on Food Perception

**DOI:** 10.3390/foods11070931

**Published:** 2022-03-24

**Authors:** Siyue Zhang, Jinzi Qian, Chenjing Wu, Dexian He, Wei Zhang, Jing Yan, Xianyou He

**Affiliations:** 1 Key Laboratory of Brain, Cognition and Education Sciences, Ministry of Education China, School of Psychology, Center for Studies of Psychological Application, and Guangdong Key Laboratory of Mental Health and Cognitive Science, South China Normal University, Guangzhou 510631, China; 2020023635@m.scnu.edu.cn (S.Z.); wuchenjing@m.scnu.edu.cn (C.W.); dexianhe@m.scnu.edu.cn (D.H.); cheungwai@m.scnu.edu.cn (W.Z.); 2020023632@m.scnu.edu.cn (J.Y.); 2 Academy of Fine Arts, South China Normal University, Guangzhou 510631, China; 2020021619@m.scnu.edu.cn

**Keywords:** aesthetic, plate pattern, plating, food and health

## Abstract

Interest has been growing in the role of subjective aesthetics in the field of food. This study explored the mechanisms by which the aesthetic appeal of plate patterns influences consumers’ perceptions of food. Three experiments were conducted to compare whether different levels of beauty and types of plate pattern aesthetics (classical versus expressive) affected the perceptions of tastiness and healthiness of the food offered. Experiment 1 was carried out with 30 participants, and the results showed that participants perceived the food presented on more beautiful plates as tastier and healthier than the food on less beautiful plates. Experiment 2 was carried out with 128 participants; the results showed that, for expressively aesthetic plates, the participants experienced more positive emotions for very beautiful plates and more negative emotions for less beautiful plates. However, for classical aesthetic plates, participants’ emotions were not affected by the beauty of the plate. Experiment 3 was carried out with 149 participants, and the results showed that, for classically aesthetic plates, participants perceived the food placed in the middle to be tastier than food placed at the edge; however, for expressively aesthetic plates, food placement did not affect participants’ perceptions of food. These results demonstrate the importance of the subjective beauty of plate patterns in influencing consumers’ food perceptions, although this influence varies depending on the type of aesthetic design of the plate pattern.

## 1. Introduction

In everyday experiences, food is never presented or served in isolation [1]. Plates are tableware that are often used for the presentation of food. Everything from the choice of the plate itself to the complex spatial arrangement upon the plate is important for the final acceptance of a dish by the diner [2]. It has long been a tradition of many cultures (Chinese, Iranian, etc.) to paint (glaze) floral patterns on plates. Producers who make plates more aesthetically pleasing certainly hope that the more beautiful plate will improve the users’ dining experience. Many studies have shown that tableware can contribute to diners’ health and overall multisensory eating experiences [3]. However, does the beauty of plates have an effect on food perceptions or plating preferences? This study examined whether and how the aesthetics of plate patterns influence perceived judgments about food.

Inspired by experimental aesthetics and visual art, plates have attracted the attention of many researchers and restaurant practitioners as important external cues to food [1]. Chefs use the plate as a canvas to enhance cooking to a full-sensory art. The way food is presented on the plate conveys a message to the consumer from the producer about the careful handling of food; consumers prefer food when it is presented in a more attractive way on the plate [4,5].

Decisions about plating have long been based on the chef’s intuition, which is often accepted by the general public; however, recent studies have found some common principles underlying plating. Consumers prefer balanced food plating rather than unbalanced plating [6]. Researchers argue that many of the principles proposed by experimental aesthetics regarding visual art perception could serve as guidelines for optimal plating [7,8,9,10,11]. The preference for balance and center location in plating was further validated by one study [12] that showed a positive association between balanced plating and pleasure, and the proximity association in the proximity-avoidance theory [13]. It is worth noting that consumer preferences for balance plating may be influenced by context [12], although this hypothesis is not supported by the results.

In addition to a preference for balance, consumers had a preference for a right ascending slant when they rotated linear dishes themselves [14,15]. However, consumers rotating their plates may be influenced by the food on the plate, as consumers aim to avoid the presence of “offensive” cues caused by the placement [16]. In a cross-cultural study of plating [17], different preferences emerged among participants from different cultures for the position of the main ingredients: American participants tended to choose a center-right position; Japanese participants tended to choose a center-bottom position; and Italians tended to place the main ingredients in the bottom-right.

Other than in the art of plating, which uses the plate as a canvas, the color and shape of the plate has also received considerable attention. Researchers have found that foods placed on round white plates are considered to be sweeter or have higher hedonic expectations, but foods placed on black plates receive higher ratings in quality and liking [18,19,20]. This may be the result of the color contrast [19]. However, some researchers have found no significant association between the color of the plate and the perceived sweetness of food [21]. Additionally, some researchers [19] have suggested that the shape of the plate has no effect on perceptions of sweetness. Moreover, the color of the plate has a significant effect on both the predicted intensity and the actual perception of spicy foods, which may indicate a cross-modal correspondence between “color-hot and warm-spicy” [22,23]. It should be noted that the impact of the plate on young people is very important. Changing the shape and size of plates could be an effective waste-reduction strategy for college meals [24], and plate color may be a key factor in determining the energy intake in young females [25].

Many studies have shown that the aesthetic features of the plate, such as the color and shape [26], as well as plating, play important roles in the consumer’s perception of a meal. However, there is still a lack of research on the effect of the aesthetics of the plate itself on the perception of the food presented on it.

Aesthetics is the study of mental processes that underlie objective evaluative experiences [26]. It defines the characteristics of beauty as a pleasurable experience [27]. In the present study, we used the word “beauty” as a subjective feeling, following the subjective rating manipulation described in a previous study [26], and used participants’ subjective ratings as a basis for distinguishing between high and low levels of beauty.

Many plates in real life have aesthetically valuable patterns (glazes), which are used as decorations (or trademarks). In this study, our plate pattern referred to glazed decorations on the plate, which have aesthetic aspects. To date, no studies have investigated how the aesthetics of plate patterns affect food perception, perhaps because the directions and mechanisms by which the attributes of beauty affect food are not obvious.

Evidence from cognitive neuroscience [28] has suggested that viewing images of delicious food activates the taste cortex of the brain, which simulates pleasing flavors. However, studies have shown that high beauty may lead to an inference that “the maker worked harder” [29] and consumers do not want to undermine the maker’s efforts; thus, the beauty of the food could have a negative impact on consumers.

One possible explanation for the relationship between beauty and other characteristics of food, such as taste and health, is a general halo effect [30,31]. However, new research suggests that more beautiful food is also considered healthy [32] because classical rather than expressive aesthetic features make it look more natural. “Classical aesthetics” [33] represents traditional aesthetic concepts (e.g., well-organized, clear, clean, and symmetrical). “Expressive aesthetics” [33] represents qualities that go beyond classical principles, emphasizing the designer’s creativity and expressiveness (e.g., originality and attractive design). According to the available studies, the effects of different beauty aesthetics on food perceptions may vary.

This study proposes that a halo effect of beauty exists, but that different types of aesthetics may play an important role in influencing the effect of beauty on food perception because of their differences in design principles.

Since the development of the generalized halo effect, the first hypothesis was that the beauty of plate patterns would significantly affect food perception. The first prediction was that if food is placed on a beautifully patterned plate, it will be perceived as tastier and healthier than food placed on a less beautifully patterned plate.

In addition, emotion is an important factor in the perceptions of beauty and food [34,35]. Therefore, the effect of aesthetically designed patterns plates on consumer emotions was a focus of this study. Expressive aesthetics conveys the designer’s personal intentions, and designers are known to have a bias for high beauty (and an aversion to low beauty) during the creative process, which is associated with their creative emotions; therefore, these emotions are likely to influence the consumer’s emotions through expressive aesthetics (high beauty triggers positive emotions/low beauty triggers negative emotions). Therefore, the second hypothesis was that the different aesthetically designed patterned plates would influence the consumer emotions in specific ways. This study predicted that if the consumer was eating from an expressive aesthetic patterned plate, it would trigger a change in the consumer’s emotions, which would mediate the effect of beauty on the perception of food. However, if the consumer was eating from a classical aesthetic patterned plate, it would not trigger a change in the consumer’s emotion, and not result in the mediation of the effect of beauty on the perception of food.

In addition, the interaction between the aesthetics of plate patterns and the plating of the food was a focus of attention. People overall prefer balanced plating [6,12,16], although the preference for balance may change with the situation (e.g., regular restaurant or fine dining). When it comes to the visual aesthetics of food, context is a key factor [6,12].

This study proposes that while consumers are eating, the pattern of the plate acts as a “mini background” as the focus is on the food on the plate. Therefore, the third hypothesis was that different aesthetic patterns of plates influence consumers’ preferences for plating. “Balance” is defined as the focus of aesthetic research on spatial composition [26], i.e., how elements are distributed around the center of a spatial frame. In this study, balance refers to the sense of balance in plating, which we define as balanced when food is placed in the center of the plate. This study proposes that expressive aesthetic patterned plates may induce a preference for novelty (unbalance) and preference for an off-center (edge) placement. In contrast, classical aesthetic designs would induce a preference for order, resulting in a preference for the central placement of food. Therefore, the prediction is that if food is placed on a beautifully expressive aesthetic pattern plate, consumers will prefer unbalanced (edge) plating, and if the food is placed on a high beauty classical aesthetic patterned plate, they will prefer balanced (center) plating.

This study used three experiments designed to explore the effect of the aesthetics (subjective beauty level) of the pattern of the plate on the perception of food and the plating preferences.

The relationship between the experiments and the hypotheses is shown in the list.

**H1**:  *The beauty of plate patterns would significantly affect food perception*.

(tested in Experiment 1)

**H2**:  *Different aesthetically designed patterned plates would influence the consumer emotions in specific ways*.

(tested in Experiment 2)

**H3**:  *Different aesthetic types patterns of plates influence consumers’ preferences for plating*.

(tested in Experiment 3)

## 2. Materials and Methods

To address the concerns of this study, as well as to test the three hypotheses proposed, three independent experiments were carried out. Experiment 1 used computerized keystroke scoring to investigate the effect of plate pattern aesthetics on subjects’ expectations of food taste and healthiness, and Hypothesis 1 was tested by comparing food perceptions in conditions with different beauty levels and different aesthetics of plate patterns. Experiment 2 used laboratory tests to conduct a field experiment of tasting food to investigate the effect of plate pattern aesthetics on subjects’ food element judgments and composite judgments, and to test Hypothesis 2 by comparing food perceptions in conditions with different beauty levels and varied plate pattern aesthetics. Meanwhile, the effect of plate pattern beauty on participants’ emotions and the mediating effect of emotions between plate pattern beauty and food perception were examined in Experiment 2. Experiment 3 followed the previous operational paradigm [36]. A field experiment of tasting food was conducted using laboratory tests to investigate the effect of food placement in different aesthetics of patterned plates on subjects’ food element judgments and composite judgments, and Hypothesis 3 was tested by comparing food perceptions under conditions of different food placements and different plate pattern aesthetics.

### 2.1. Experiment 1

#### 2.1.1. Study Design

Experiment 1 used a 2 (beauty level of the plates: high/low) × 2 (aesthetics of the plate patterns: classical aesthetic/expressive aesthetic) within-subject design, with the participants’ ratings of tastiness and healthiness of the food as dependent variables.

#### 2.1.2. Participants

According to G*Power calculations, the experiment required at least 22 participants to reach the medium effect size (1 − *β* = 0.8; *α* = 0.05). A total of 30 participants (14 male, 16 female) aged between 18 and 26 years (*M* = 21.80, *SD* = 2.52) took part in the study. All subjects were randomly recruited through the Subject Recruitment Information Group of South China Normal University. They conducted a 15 min experiment in the laboratory of the School of Psychology, South China Normal University. The participants gave informed consent, and reported no hearing or visual impairments. All participants were current students at the South China Normal University and were not trained. This experiment was approved by the Ethical Committee of South China Normal University.

#### 2.1.3. Materials

The process of choosing the plate materials was as follows. First, a graduate student in Art from South China Normal University was invited to design 146 plate patterns with different levels of beauty, including 102 plate patterns based on classical aesthetics and 44 patterns based on expressive aesthetics. The artist was not told the purpose of the experiment, and was told to develop the designs based on the principles of classical aesthetics and expressive aesthetics, as well as their own aesthetic judgment. The colors used for the plate patterns were all blue (78% lightness, 25% saturation, 221 hues), as commonly found on the market, all plates had a white background, and all images were 800 × 800 pixels in size. In this study, a pre-experiment was conducted to distinguish between high and low beauty levels of plate patterns. This pre-experiment was performed online, and participants were asked to rate the degree of beauty of the plate pattern on a 7-point scale. A total of 62 subjects (28 males, 34 females, *M* = 26.59, *SD* = 8.43) were recruited to evaluate the classical aesthetic patterns, and 50 subjects (20 males, 30 females, *M* = 24.36, *SD* = 5.58) evaluated the expressive aesthetic patterns. The beauty level reflected the subjective aesthetic judgments of the pattern. A rating of “1” indicated that the pattern in the image was not at all beautiful, whereas “7” indicated that the pattern in the image was very beautiful. Finally, we selected the top five images with the highest beauty level ratings for each aesthetic as the beautiful patterns and the top five images with the lowest beauty level ratings as the less beautiful patterns. The beauty rating of the plates with the highest beauty ratings (*M* = 5.33, *SD* = 0.88) was significantly higher than that of the plates with the lowest beauty ratings (*M* = 2.713, *SD* = 0.97), *t*_(19)_ = 22.52, *p* < 0.001, *Cohen’s d* = 2.79.

During the experiment, the images viewed by the subjects were presented as a combination of the chocolate and plate. Pictures of chocolate were chosen from two different types of chocolate from “Daily Dark” (chocolate brand), as actual pictures of chocolate and adjusted versions of chocolate.

**Figure 1:**
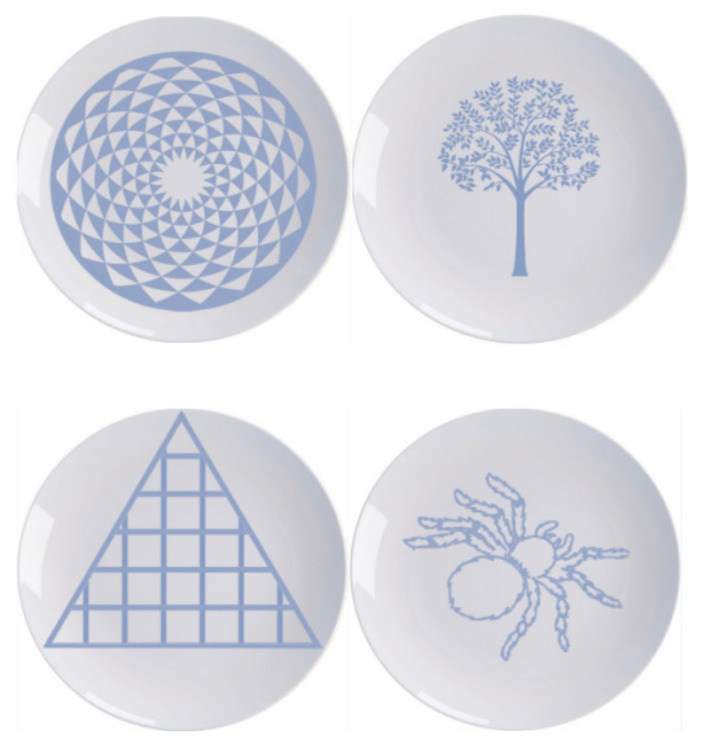
Example of patterned plates. The classical aesthetic patterned plates are on the left, and expressive ones on the right, with high beauty on the top and low beauty on the bottom.

Experiment 1 presented a combination of chocolate and a patterned plate; to rule out the possibility that the complexity of the resulting combination image affected the results, we performed a complexity rating study. For this study, we recruited 39 subjects (21 males and 18 females, *M* = 21.38, *SD* = 2.24) through an online platform and asked them to rate the complexity of each picture of the plate with chocolate (as in the experiment) on a 7-point scale. A score of “7” indicated “very complex”, and “1” indicated “very simple”. The results found no significant difference between high beauty and low beauty stimuli in terms of complexity (*t*_(38)_ = 1.008, *p* > 0.05).Examples of patterned plate of the experimental materials are shown in Figure 1

#### 2.1.4. Procedure

**Figure 2:**
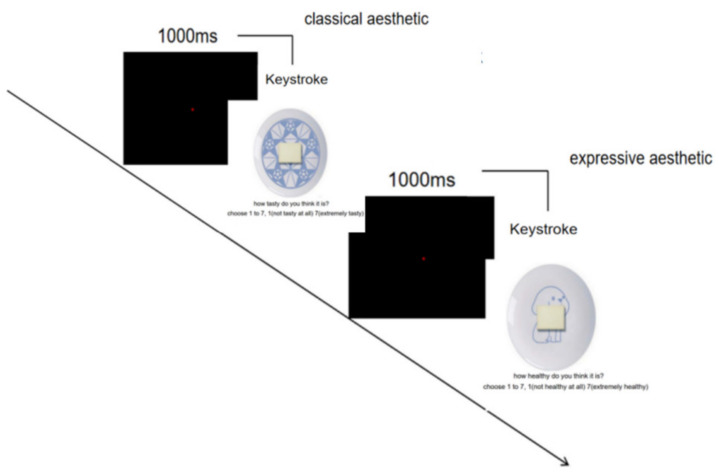
Flow chart of Experiment 1.

The experiment was conducted in the psychological laboratory of South China Normal University. The lab provided participants with a seat, a table, and a computer, with no additional variables that could interfere with attention. Experiment 1 was programmed using Eprime 2.0. Participants were asked to view a combined picture of a plate with food presented on a computer screen and to rate the tastiness (how tasty do you think it is?) and healthiness (how healthy do you think it is?) of the food in the picture on a 7-point scale, with “1” being not (tasty/healthy) at all and “7” being extremely (tasty/healthy). Before starting the main task, participants completed practice trials for task familiarization. After the exercise, participants began the formal experiment. In the experiment, the classical aesthetic patterned plate and expressive aesthetic patterned plate were presented in different blocks; participants were presented with all pictures and asked to rate the tastiness and healthiness of the food in the picture, with the beautiful and less beautiful plates presented in a random order. Halfway through the experiment, participants had a 5 min break. The experiment lasted approximately 15 min. The procedure of the experiment is shown in Figure 2.

### 2.2. Experiment 2

#### 2.2.1. Study Design

Experiment 2 used a 2 (beauty of the plates: high beauty/low beauty) × 2 (aesthetics of the plate patterns: classical aesthetic/expressive aesthetic) between-subjects design, with the participants’ rating of tastiness, sweetness, greasiness, pleasant aroma, spicy aroma, nutritional content, benefits to the body, caloric content, and price estimation of chocolate as dependent variables.

We focused on four dependent variables examining elemental features involving taste and smell, sweetness, greasiness and pleasant aroma, and spicy aroma, as adopted by Kpossa and Lick [20] and Apaolaza et al. [31]. These four dimensions are in line with the definition from Stewart and Goss [18] of elemental evaluations, i.e., these dimensions all belong to “the basic judgments of some individuals”. With these four dependent variables, we aimed to investigate the effect of different plate patterns, i.e., classical aesthetics and expressive aesthetics, on elemental perceptions.

We also examined three compound judgments of tastiness, healthiness, and price estimation. These three dependent variables have been the focus of attention in previous studies in the literature [4,5,36,37], because they are more representative of the overall consumer experience of food. Regarding healthiness, we followed Hagen’s [32] definition of perceived healthiness as comprising a complex of high-benefit, high-nutrition, and low-calorie concepts, which is consistent with the current dietary guidelines for understanding health. We conducted a factor analysis of benefit, nutrition, and calories (reverse-coded) and synthesized healthiness scores based on component loadings.

The purpose was to examine the effect of different plate pattern beauty levels and aesthetics on composite perceptions using these three dependent variables.

#### 2.2.2. Participants

According to G*Power calculations, the experiment required at least 112 participants to reach the medium effect size (1 − *β* = 0.8; *α* = 0.05). A total of 136 (100 females, 36 males) aged between 18 and 26 years of age (*M* = 20.96, *SD* = 2.25) took part in the study. All subjects were randomly recruited through the Subject Recruitment Information Group of South China Normal University. They conducted a 10 min experiment in the laboratory of the School of Psychology, South China Normal University. The participants gave informed consent and reported no hearing or visual impairments. All the participants were current students at the South China Normal University and were not trained. This experiment was approved by the Ethical Committee of South China Normal University.

#### 2.2.3. Materials

In total, four physical plates were used in Experiment 2, with a single plate for each of the conditions (beautiful classical aesthetic patterned plate, less beautiful classical aesthetic patterned plate, beautiful expressive aesthetic patterned plate, and less beautiful expressive aesthetic patterned plate). The distinctions between high and low beauty scores were obtained from the prior online questionnaire ratings. No significant difference was noted in the beauty rating between the classical aesthetic patterned high (low) beauty plate and the expressive aesthetic patterned high (low) beauty plate. The size of all of the plates was 18.3 cm in diameter and the patterns used were presented in common blue colors available on the market (78% lightness, 25% saturation, 221 hues). The materials used were all ceramic, custom-made by Youneng Ceramic Technology (Organization code MA4ULXDK2).

**Figure 3:**
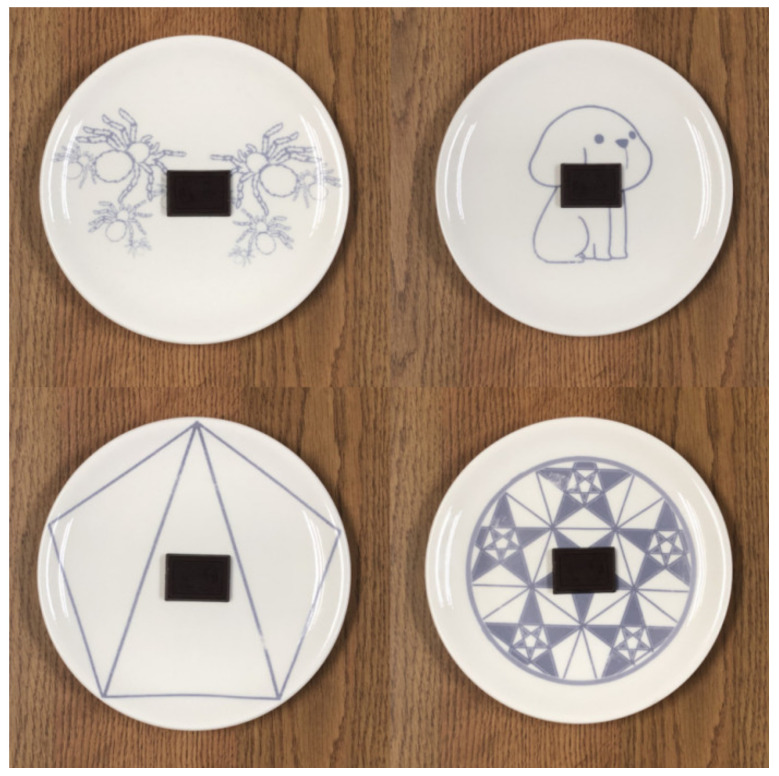
Example of expressive aesthetic pattern plates (**top**) and classical aesthetic pattern plates (**bottom**) combined with the chocolate. The plates on the right side were both rated as having a high beauty level and those on the left were rated as having a low beauty level.

In the experiment, each participant was asked to taste a 10 g piece of salted-caramel-flavored dark chocolate (Figure 3), produced by the commercially available brand Daily Dark Chocolate (licensed by Landbaes Internation-Alcd, Ltd., Cardiff, UK), placed on a plate. Each participant was given a list of the recipe ingredients and the nutritional composition of the chocolate and was told that they needed to eat the whole piece of chocolate; otherwise, they would be considered as not having completed the experiment.

#### 2.2.4. Procedure

The experiment was conducted in the psychological laboratory of South China Normal University. The lab had a table, a chair, and a box of disposable gloves and tissues on the table. There were no additional factors that might have influenced the participants. In this study, participants were numbered in the order of online registration experiment, and randomly assigned to four experimental groups using patterned plate with beauty level (high/low) × aesthetics (classical aesthetics/expressive aesthetics). Participants were provided with the plate they were about to use at the beginning of the experiment and were asked to look at the plate for 30 s to ensure that they observed the pattern. Then, the participants were asked to complete a PANAS mood scale [38]. In the PANAS mood scale, a more positive mood is associated with happiness, and a more negative mood is associated with anxiety [39].

After completing the questionnaire, the participants were provided with detailed nutritional composition of the food they were about to taste and told by the experimenter that they needed to read it carefully for 30 s; this was to prevent the participants from having doubts about the safety of the food. After watching, their plates were taken back by the experimenter. After the experimenter had placed the chocolate on the plate, both the plate and the chocolate were given to the participants. Participants were told to eat the food in their usual way and to try and eat it all.

After eating the chocolate, the participants completed the questionnaire. The items on the questionnaire included ratings of tastiness, sweetness, greasiness, pleasant aroma, spicy aroma, nutritional content, benefits to the body, caloric content, and price estimation on a scale of 1–7, with “1” being not at all and “7” being extremely. The experiment lasted approximately 8 min.

The contents of the questionnaire were as follows:

How tasty do you think the dessert you just ate was?
How sweet do you think the dessert you just ate was?
How greasy do you think the dessert you just ate was?
How much of a pleasant aroma do you think the food you just ate had?
How much of a spicy aroma do you think the food you just ate had?
How much nutritional content do you think the food you just ate had?
How much do you think the food you just ate is beneficial for your body?
How caloric do you think the food you just ate was?
Please estimate the price of the food you just ate.

### 2.3. Experiment 3

#### 2.3.1. Study Design

Experiment 3 used a 2 (placement of food: center/edge) × 2 (aesthetics of the plate patterns: classical aesthetic/expressive aesthetic) between-subjects design, with the dependent variable being the participants’ ratings of tastiness, sweetness, greasiness, pleasant aroma, spicy aroma, nutritional content, benefits to the body, caloric content, and price estimation of chocolate on the plates.

#### 2.3.2. Participants

According to G*Power calculations, the experiment required at least 112 participants to reach the medium effect size (1 − *β* = 0.8; *α* = 0.05). A total of 149 valid participants (114 female, 35 male) aged between 18 and 27 years of age (*M* = 21.57, *SD* = 2.03) took part in the study. All subjects were randomly recruited through the Subject Recruitment Information Group of South China Normal University. They conducted a 10 min experiment in the laboratory of the School of Psychology, South China Normal University. The participants gave informed consent, and reported no hearing or visual impairments. All participants were current students at the South China Normal University and were not trained. This experiment was approved by the Ethical Committee of South China Normal University.

#### 2.3.3. Materials

**Figure 4:**
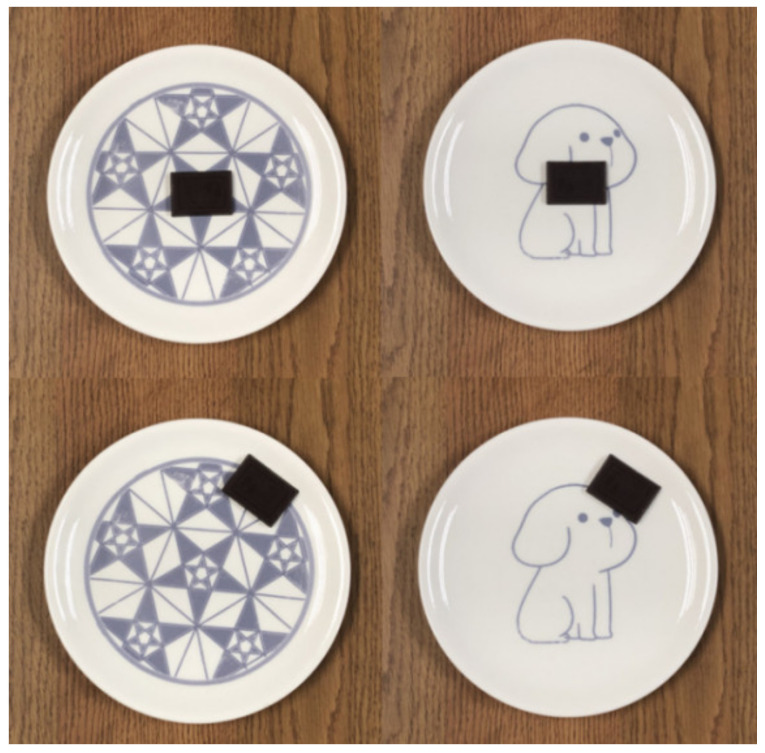
Example of a beautiful classical aesthetic pattern plate (**left**) and a beautiful expressive aesthetic pattern plate (**right**) with the chocolate at the center (**top**) and edge (**bottom**).

Two physical plates were used in Experiment 3: a beautiful classical aesthetic pattern plate and beautiful expressive aesthetic pattern plate. In the experiment, each participant was asked to taste a 10 g piece of salted-caramel-flavored dark chocolate (Figure 4), produced by the commercially available brand Daily Dark Chocolate, and served on a plate. Each participant was given the recipe composition list of the chocolate, as well as the nutritional composition list, and was informed that they needed to finish the whole piece of chocolate or they would be considered as not having completed the experiment.

#### 2.3.4. Placement

This experiment drew on the findings of Michel et al. [36], who distinguished between the central and edge placement of food (chocolate). In this experiment, central placement was defined as “balance” and edge placement as “unbalance”.

A pilot test was conducted to test this manipulation, using a balance rating scale of 1–7, with “1” being not balanced at all and “7” being very balanced. The pilot test was conducted online with 38 participants (21 males, 19 females). The results showed that there was no difference between the perceived balance of the central arrangement of the classical aesthetic patterned plate (*M* = 5.74, *SD* = 1.75) and perceived balance of the central arrangement of the expressive aesthetic patterned plate (*M* = 5.32, *SD* = 1.65); there was no difference between perceived balance of the edge arrangement of the classical aesthetic patterned plate (*M* = 2.34, *SD* = 1.79) and perceived balance of the edge arrangement of the expressive aesthetic patterned plate (*M* = 2.58, *SD* = 1.46). The difference between the perceived balance of the central arrangement of the plates and the edge arrangement was significant for both plates (classical aesthetic patterned plate group, *t*_(37)_ = 7.970, *p* < 0.001, *Cohen’s d* = 1.92; expressive aesthetic patterned plate group, *t*_(37)_ = 12.503, *p* < 0.001, *Cohen’s d* = 1.76).

#### 2.3.5. Procedure

The experiment was conducted in the psychological laboratory of South China Normal University. The lab had a table, a chair, and a box of disposable gloves and tissues on the table. There were no additional factors that might have influenced the participants. In this study, participants were numbered in the order of online registration experiment, and randomly assigned to four experimental groups of using patterned plate with food placement (center/edge) × aesthetics (classical aesthetics/expressive aesthetics). The participants were provided with the plate they were about to use at the beginning of the experiment, and were asked to look at the plate for 30 s to ensure that they had observed the pattern on the plate. Then, they were provided with details of the nutritional composition of the food they were about to taste, and told by the experimenter that they needed to read it carefully for 30 s; this was to prevent the participants from having doubts about the safety of the food. After watching, the plates were be taken back by the experimenter. After the experimenter had placed the chocolate on the plate, both the plate and the chocolate were given to the participants. Participants were told to eat the food in their usual way and to try to eat it all.

### 2.4. Data Analyses

For several pre-experiments, we used independent samples *t*-tests to test whether there were differences between the experimental variables (beauty, complexity, and balance).

For Experiment 1, we used a repeated measures ANOVA with both the level of beauty of the plate pattern and the aesthetics as within-subject variables. After further results were obtained, we conducted a simple effects analysis of interaction.

For Experiment 2, we used an ANOVA with both the level of beauty of the plate pattern and the aesthetics as between-subjects variables. After further results were obtained, we conducted a simple effects analysis of interaction. To assess the mediating effect of emotions, we conducted simple mediation analysis using the PROCESS macro for SPSS (model 4; 5000 samples).

For Experiment 3, we used an ANOVA with both the aesthetics of the plate pattern and the food placement (plating) as between-subjects variables. After further results were obtained, we conducted a simple effects analysis of interaction.

All statistical analyses were performed using SPSS Statistics (Version 23, IBM Corporation, New York, NY, USA) at a 5% level of significance.

## 3. Results and Discussion

### 3.1. Experiment 1

MANOVA results (Table 1) showed that the main effect of the beauty level of the plate pattern was significant for both the tastiness rating and healthiness rating. The aesthetics of the plate pattern had no significant effect on food expectations. However, the interaction between the beauty level of the plates and the aesthetics of patterns was significant for both the tastiness rating and healthiness rating.

**Figure 5:**
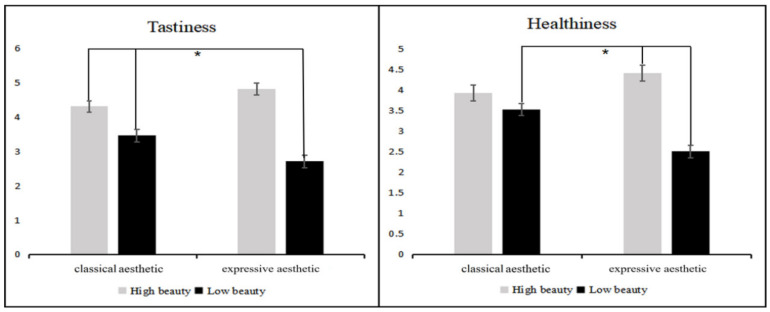
Tastiness scores (**left**) and healthiness (**right**) for plates with different pattern types and different beauty levels. Error lines represent 95% confidence intervals. Significant differences (*) are indicated between the connecting lines.

The simple effects analysis (Figure 5) revealed that tastiness scores were significantly lower for the less beautiful expressive aesthetic pattern plates than for all other plates (vs. beautiful classical aesthetic patterned plate, *p* < 0.001; vs. beautiful expressive aesthetic patterned plate, *p* < 0.001; vs. less beautiful classical aesthetic patterned plate, *p* < 0.05). However, for food presented on the beautiful expressive aesthetic pattern plates, participants scored tastiness significantly higher than almost all other plates (not significantly higher than the beautiful classical aesthetic patterned plate, *p* *>* 0.05). Notably, within the classical aesthetic group, tastiness scores were likewise significantly higher for beautiful patterned plates than for low beauty patterned plates (*p* < 0.05).

The expected scores for healthiness appeared as similar to the expected scores for tastiness. Healthiness scores were significantly lower for the less beautiful expressive aesthetic pattern plates than for all other meal plates (all comparisons, *p* < 0.001). However, for food presented on the beautiful expressive aesthetic pattern plates, participants scored healthiness expectations significantly higher than almost all other plates (not significantly higher than the high beauty of classical aesthetic patterned plate, *p* > 0.05).

### 3.2. Experiment 2

The results of the ANOVA are shown in Table 2 and Table 3.

#### 3.2.1. Element Judgment

The results of the experiment showed a significant main effect of the beauty of the plate on the participants’ rating of taste aspects (both sweetness and greasiness), but not on ratings of significant effects on the olfactory aspects. The aesthetic pattern (classical versus expressive) had no significant effect on the participants’ elemental judgments. However, there was a significant interaction between the beauty of the plate pattern and the aesthetic pattern type in the assessment of spicy aroma.

**Figure 6:**
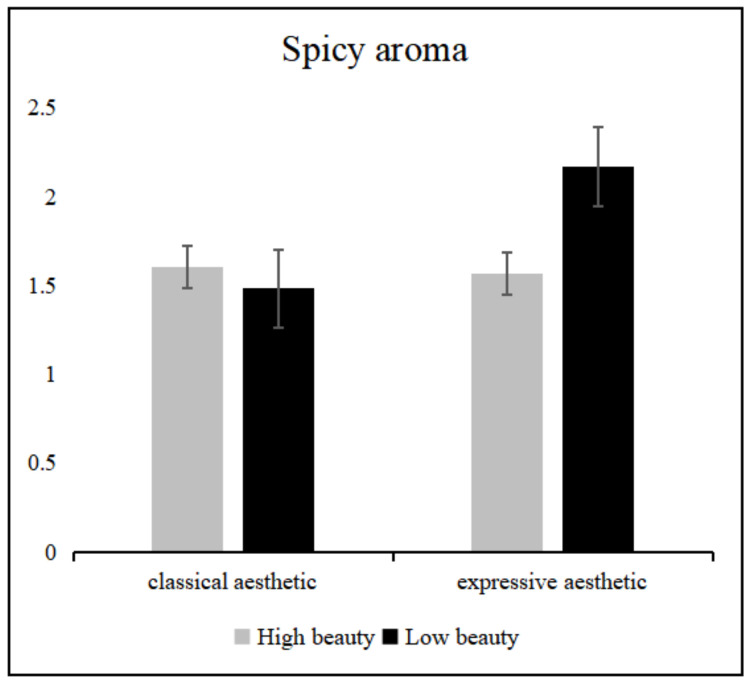
Spicy aroma scores for different pattern types with different beauty levels of plates. Error lines represent 95% confidence intervals.

The simple effects analysis showed (Figure 6) that, for the rating of spicy aroma, the less beautiful expressive aesthetic patterned plate group rated it significantly higher than all other plate groups (vs. beautiful classical aesthetic patterned plate, *p* < 0.05; vs. beautiful expressive aesthetic patterned plate, *p* < 0.05; vs. less beautiful classical aesthetic patterned plate, *p* < 0.05). There was no significant difference between the other plate groups for spicy aroma ratings.

#### 3.2.2. Compound Judgment

There was a significant main effect of the beauty of patterned plates on the participants’ ratings of tastiness, but not on the ratings of healthiness (Table 3). The aesthetic pattern type had no significant effect on any of the compound judgments. However, there was a significant interaction between the beauty of the plate pattern and the aesthetics for price estimation.

**Figure 7:**
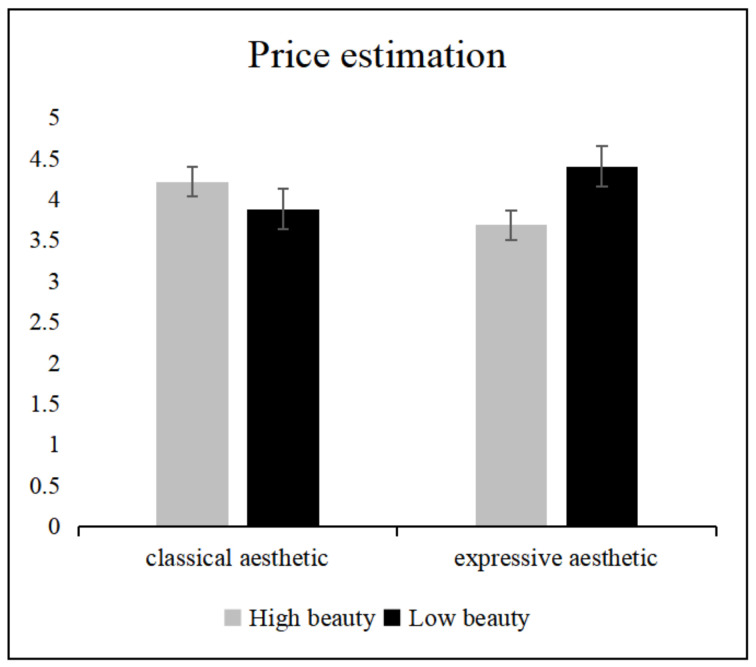
Price estimation for plates with different pattern types and different beauty levels. Error lines represent 95% confidence intervals.

The simple effects analysis revealed (Figure 7) that, for price estimation, participants assessed the price of food presented on the less beautiful expressive aesthetic pattern plate significantly higher than food presented on the beautiful expressive aesthetic pattern plate (*p* < 0.05). There were no significant differences in price estimations between the other plates.

#### 3.2.3. Emotion

Emotions were the focus of attention in Experiment 2. The results showed a significant interaction between the two independent variables of beauty level and aesthetics for both positive emotions (*F*(1,132) = 6.050, *p* < 0.05, *η*^2^ = 0.044) and negative emotions (*F*(1,132) = 7.225, *p* < 0.05, *η*^2^ = 0.052).

**Figure 8:**
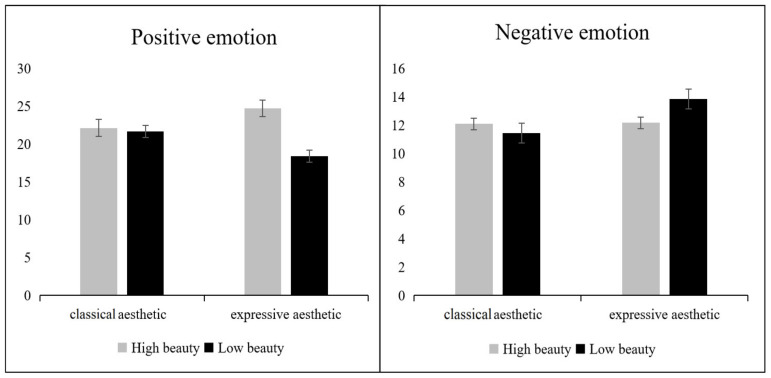
Positive emotions (**left**) and negative emotions (**right**) for different pattern types with different beauty levels of plates. Error lines represent 95% confidence intervals.

The simple effects analysis (Figure 8) showed that, for the positive emotion dimension, the less beautiful expressive aesthetic pattern plate scored significantly lower than all other plates (vs. beautiful classical aesthetic patterned plate, *p* < 0.05; vs. beautiful expressive aesthetic patterned plate, *p* < 0.001; vs. less beautiful classical aesthetic patterned plate, *p* < 0.05). In the negative emotion dimension, the less beautiful expressive aesthetic plate scored significantly higher than all other plates (vs. beautiful classical aesthetic patterned plate, *p* < 0.05; vs. beautiful expressive aesthetic patterned plate, *p* < 0.001; vs. less beautiful classical aesthetic patterned plate, *p* < 0.05). There were no differences in negative emotion scores between any of the other plate groups.

**Figure 9:**
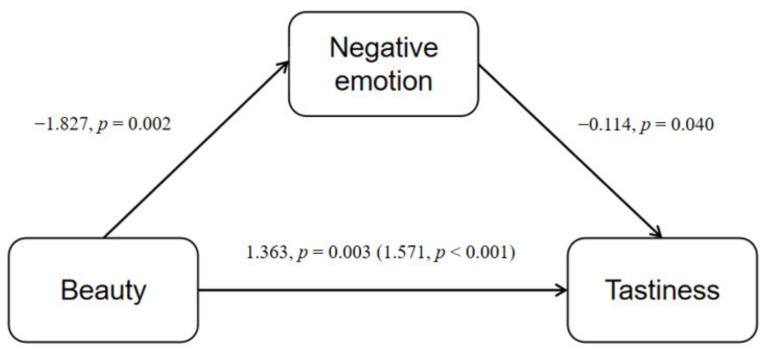
Mediation of the relationship between beauty and tastiness by negative emotion. Analysis was limited to participants in the expressive aesthetic group.

Only participants in the expressive aesthetics patterned plate group showed a significant effect of emotion; therefore, our analysis of the effect of emotion was performed only within the expressive aesthetics group. A regression analysis showed that positive emotion scores were significantly positively correlated with tastiness B = 0.088, β = 0.321, *p* < 0.05), whereas negative emotions were only significantly negatively correlated with tastiness (B = −0.172, β = −0.342, *p* < 0.05). We conducted simple mediation analysis using the PROCESS macro for SPSS (model 4; 5000 samples); a significant partial mediation effect of negative emotions was shown in the dimension of tastiness (*p* < 0.05). The mediating effect of emotions (both positive and negative) was not significant for any other dimension (Figure 9; Table 4).

### 3.3. Experiment 3

The results of the ANOVA are shown in Table 5.

#### 3.3.1. Elemental Judgments

Ratings on all dimensions were not significantly influenced by the placement of the food on the plate (perceived balance) or by the aesthetics of the plate.

#### 3.3.2. Compound Judgments

There was a significant main effect of food placement (perceived balance) on the participants’ ratings of tastiness and healthiness, but not on price. There was a significant interaction between the aesthetics of plate patterns and food placement locations in the tastiness and healthiness rating dimensions.

**Figure 10:**
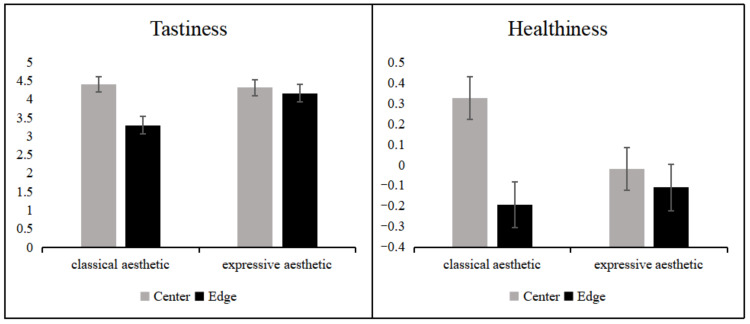
Tastiness (**left**) and healthiness (**right**) for differently patterned plates with different placements of food. Error lines represent 95% confidence intervals.

The simple effects analysis revealed (Figure 10) that consumers perceived food placed in the center of the classical aesthetic plate to be significantly tastier (*p* < 0.05) and healthier (*p* < 0.05) than food placed at the edges of the classical aesthetic plate. In particular, participants perceived food placed in the center of the classical aesthetic plate to be healthier than any other plate and placement (vs. food placed in the edge of the classical aesthetic plate, *p* < 0.05; vs. food placed in the center of the expressive aesthetic plate, *p* < 0.05; vs. food placed in the edge of the expressive aesthetic plate, *p* < 0.05).

However, the food placement did not make a difference in terms of the participants’ perceptions of tastiness and healthiness on the expressive aesthetic plate patterns.

#### 3.3.3. Price Estimation

The price estimation replicated the results of Experiment 2, with a significant main effect on the aesthetics. Participants perceived the food placed on expressive aesthetic pattern plates (corresponding to the beautiful expressive aesthetic pattern plate of Experiment 2) to be lower in price than food placed on the classical aesthetic pattern plate.

### 3.4. Discussion

This study demonstrated, for the first time, that the level of beauty and the type of aesthetics of a plate pattern had different effects on food perceptions. Experiment 1 validated previous theories proposing that beauty acted through a generalized halo. As an example, in one study, beautiful apples were perceived to be more delicious and possess other positive qualities [40]. However, our further study showed that beauty affects food perception not only in the form of halo effects: different aesthetic designs have different effects on consumers’ food perception. In Experiment 2, we tested the hypothesis that only the expressive aesthetics plate pattern affected consumers’ emotions (the classical aesthetics plate pattern did not affect emotions) and tested the mediating effect of emotions on food perception within the expressive aesthetics plate pattern group. Experiment 3 verified the hypothesis that only classical aesthetic plate patterns showed a benefit from balance (central) presentation.

Regarding emotions, Rizzato et al. [41] showed that food perception was influenced by emotions, although these emotions are not necessarily food-related. Kantono et al. [42,43] showed that emotions induced by music significantly influenced taste perceptions (positive emotions were associated with sweetness and negative emotions with bitterness). Our experiments did not replicate this result well for elemental judgments. In terms of composite judgments, our results only showed that tastiness was positively correlated with positive emotions and negatively correlated with negative emotions. This may be because we elicited emotions in participants in a different way from that used in previous studies. The source of the emotional effect on consumers in Experiment 2 is thought to have been an emotional change in the creation process of the plate designer, which was conveyed in the form of a patterned plate designed on the basis of an expressive aesthetic definition. This again suggests that the principles of visual art perception are applicable in food science. Our hypothesis of an emotion-mediated effect has received conclusive support: the mediating effect of negative emotions on tastiness ratings was significant. However, we did not observe any mediating effect of positive emotions. We propose that the reason for this is, on the one hand, that the direct effect of beauty on tastiness scores is strong, and, on the other hand, because we used scales to divide participants’ emotions into positive and negative dimensions, although, in real life, consumers’ emotions affect food perception in a holistic way.

For balanced plating, many previous studies have shown that consumers prefer balanced and centrally located plating. This result was again observed for the beautiful classical aesthetic plate group in Experiment 3. In the balanced (center) condition, participants rated all three dimensions of tastiness and healthiness (composite judgment) and sweetness (elemental judgment) significantly higher than in the unbalanced (edge) condition. More importantly, however, for the first time, our experimental results validated the hypothesis that balance preferences for plating were influenced by the context in which the food is served, as hypothesized by Velasco et al. [6]. The results of Experiment 3 showed that the perception of food in beautiful expressive aesthetic patterned plates was not influenced by perceived balance (placement). However, we did not find our hypothesized relationship of a preference for more novel (dissonant) placements among participants using highly aesthetically expressive aesthetic plates.

Regarding price estimations, we obtained unexpected results, where chocolate on expressive aesthetic plates with low beauty scores received higher price estimates than those with high beauty scores. We suggest that food neophilia may play a role: consumers who are neophilics will be more receptive to novel colored foods, such as blue potatoes [44]. Although the application of this phenomenon already exists on the Chinese market (such as restaurants with plates painted with ants, and Tang Palace tea desserts), its original intention was to imitate the artistic design of Chitins Glossby Evelyn Bracklow. Additionally, the beautiful expressive aesthetic pattern used a design (puppy) that may commonly be associated with low prices. Many snack products in the Chinese market targeted towards children use puppy-like patterns as labels (because they are cute); these products tend to be low-priced because of their small package size and target consumer group. Consumers with little relevant knowledge [45] will infer prices in stores based on the low-priced products that are easily recalled. We suggest that puppy motifs may also induce availability heuristics among participants in this way, leading to lower ratings of chocolate prices.

This research originated from the concept of classifying aesthetics [32]. The results of this experiment have some practical implications for restaurant operators because we offer the possibility of changing consumer perceptions by changing the beauty of the plate in a way that does not affect the food itself, rather than applying other methods such as additives and seasonings that may bring cost consumption and consumer health effects. We provide suggestions for including the plate patterns in the science of plating. Especially when the plate has a pattern, the plate as part of the dining environment has an important impact on the consumer’s perception.

## 4. Conclusions

This experiment empirically demonstrated the effect of aesthetic factors of plate patterns on food perception. The generalized halo induced by this aesthetic appeal should be significant and stable both during visual viewing alone and during actual eating. At the same time, this study shows that the different aesthetics of patterned plates have different effects on consumers’ food perceptions. In terms of emotion, the beauty of classical aesthetic plates does not affect consumers’ emotions, whereas the beauty of expressive aesthetic patterned plates does affect consumers’ emotions. In terms of plating, consumer preferences for balanced and central plating emerged in classical aesthetic patterned plates, but food in expressive aesthetic patterned plates was not influenced by the placement of it.

In addition, there were certain operational deficiencies to this study. Although chocolate, which is often used as a food material in many studies, was selected, there were no additional food types examined in the experiment, and the chocolate used in the experiment was a retail product (not usually consumed on a plate), which may have led to a decrease in validity. Moreover, the study did not ask participants to evaluate the beauty level of the plate patterns again in the main task. Due to the subjectivity in judging beauty, their evaluations could differ from the previous assessments. In addition, in Experiments 2 and 3, we gave participants a list of food ingredients and asked them to read it before eating in order to prevent participants from having doubts about the safety of the food. This likely resulted in the reduction in or elimination of the effects regarding perceived healthiness. In addition, the experiments were conducted in the laboratory, and it is unknown whether the same effects would be found in real restaurants.

Future studies should examine the effects of placing different types of food on patterned plates. Food consumption is an extremely crucial and practical factor for restaurant operators and consumers. The effects of aesthetic factors and emotions on food consumption are key concerns for the food industry [29,46]. Future research should attempt to address the question of whether the aesthetic factors of plate patterns affect food consumption.

## Figures and Tables

**Table 1:** Repeated measures ANOVA in Experiment 1.

Variables	Factors	F	*p*	*η* ^2^
Tastiness	Beauty	**74.598**	**<0.001**	**0.720**
	Pattern type	0.504	0.483	0.017
	Beauty × Pattern type	**18.769**	**0.002**	**0.393**
Healthiness	Beauty	**47.756**	**<0.001**	**0.622**
	Pattern type	3.870	0.059	0.118
	Beauty × Pattern type	**19.868**	**<0.001**	**0.407**

Note: (*n* = 30). For the different tests, DFn = 1 and DFd = 28. Bolded indicates statistically significant.

**Table 2:** ANOVA for element judgment in Experiment 2. (*n* = 136).

Variables	Factors	*F*	*p*	*η* ^2^
Sweetness	Beauty	**9.98**	**0.002**	**0.070**
	Pattern type	0.83	0.365	0.006
	Beauty × Pattern type	1.58	0.211	0.012
Greasiness	Beauty	**4.99**	**0.027**	**0.036**
	Pattern type	0.70	0.405	0.005
	Beauty × Pattern type	0.03	0.868	<0.001
Pleasant aroma	Beauty	0.478	0.490	0.004
	Pattern type	0.71	0.401	0.005
	Beauty × Pattern type	1.74	0.190	0.013
Spicy aroma	Beauty	1.84	0.177	0.014
	Pattern type	3.14	0.067	0.025
	Beauty × Pattern type	**4.18**	**0.043**	**0.031**

Note: (*n* = 136). For the different tests, DFn = 1 and DFd = 134. Bolded indicates statistically significant.

**Table 3:** ANOVA for compound judgment in Experiment 2.

Variables	Factors	*F*	*p*	*η* ^2^
Tastiness	Beauty	**26.58**	**<** **0.001**	**0.168**
	Pattern type	0.56	0.455	0.004
	Beauty × Pattern type	1.31	0.254	0.010
Price estimation	Beauty	0.97	0.326	0.007
	Pattern type	0.03	0.955	<0.001
	Beauty × Pattern type	**6.72**	**0.011**	**0.048**
Healthiness	Beauty	3.14	0.079	0.028
	Pattern type	2.94	0.089	0.022
	Beauty × Pattern type	0.92	0.339	0.007

Note: (*n* = 136). For the different tests, DFn = 1 and DFd = 134. Bolded indicates statistically significant.

**Table 4:** Mediation of the relationship between beauty and tastiness by negative emotion.

Mediation	B	β	SE	LLCI	ULCI	*p*
Beauty	1.363	0.412	0.358	0.648	2.078	0.003
Negative emotion	−0.114	−0.227	0.054	−0.223	−0.052	0.040

**Table 5:** ANOVA for compound judgment in Experiment 3 (*n* = 149). For the different tests, DFn = 1 and DFd = 147. Bolded indicates statistically significant.

Variables	Factors	*F*	*p*	*η* ^2^
Tastiness	Pattern type	2.92	0.090	0.020
	Placement	**7.74**	**0.006**	**0.051**
	Pattern type × Placement	**4.43**	**0.037**	**0.030**
Price estimation	Pattern type	**6.19**	**0.014**	**0.041**
	Placement	0.09	0.767	0.001
	Pattern type × Placement	0.34	0.561	0.002
Healthiness	Pattern type	1.57	0.212	0.011
	Placement	**8.39**	**0.004**	**0.055**
	Pattern type × Placement	**4.16**	**0.043**	**0.028**

## Data Availability

The data presented in this study are available on request from the corresponding author. The data are not publicly available due to restrictions e.g., privacy or ethical.
